# Supplementary material for: Recent Molecular Characterization of Porcine Rotaviruses Detected in China and Their Phylogenetic Relationships with Human Rotaviruses
Source: Viruses. 2024 Mar 14;16(3):453. doi: 10.3390/v16030453 (PMC10975774; doi:10.3390/v16030453)
Supplement: Supplementary file 1 [file viruses-16-00453-s001.zip › Supplementary Materials Table S2.pdf]

Table S2. Homology analysis of PoRVA identified in this study

			VP7							VP4							VP6						
Porcine RVA strains identified in this study	Time	Site	Closely related strains	Accession NO.	Country	Host	Year	Nucleotide similarity (%)	Accession NO. sequenced in this study	Closely related strains	Accession NO.	Country	Host	Year	Nucleotide similarity (%)	Accession NO. sequenced in this study	Closely related strains	Accession NO.	Country	Host	Year	Nucleotide similarity (%)	Accession NO. sequenced in this study
CHN/Shaanxi/HY1/2022	2022.1	Shaanxi	SCCD-A(G9)	MH910068.1	China	Dog	2017	97.96	OQ743854	MZ-MPT-192(P13)	MT784813.1	Mozambique	Pig	2016	91.88	OQ799662	TA-4-1(I5)	KT820772.1	China	Pig	2016	96.57	OQ799756
CHN/GX/XS1/2022	2022.1	Guangxi	CU-B1738(G4)	KT007665.1	Thailand	Human	2013	96.63	OQ743848	GHA-14(P7)	MN102368.1	UK	Pig	2016	93.37	OQ799656	CMH-N016-10(I1)	MG781035.1	Thailand	Human	2010	96.57	OQ799746
CHN/GD/XC3/2022	2022.1	Guangdong	D152(G9)	KU740013.1	Taiwan-China	Human	2015	94.35	OQ743851	HLJ/15(P23)	KU886316.1	China	Pig	2016	96.75	OQ799659	HN03(I5)	MH021179.1	China	Pig	2018	97.57	OQ799753
CHN/NN/XS1/2022	2022.1	Guangxi	Arg720A(G12)	EU496257.1	Argentina	Human	2009	96.95	OQ743850	CN127(P7)	ON989016.1	China	Pig	2021	96.94	OQ799658	/	/	/	/	/	/	/
CHN/NN/XS/2022	2022.1	Guangxi	VNM-NT0073(G9)	LC095899.1	Vietnam	Human	2007	97.06	OQ743849	SCYA-H2(P13)	MT198757.1	China	Pig	2019	97.06	OQ799657	HN03(I5)	MH021179.1	China	Pig	2018	98.41	OQ799751
CHN/GD/DD2/2022	2022.1	Guangdong	/	/	/	/	/	/	/	/	/	/	/	/	/	/	HN03(I5)	MH021179.1	China	Pig	2018	98.41	OQ799749
CHN/GD/DD3/2022	2022.1	Guangdong	DZ-2(G5)	KT820775.1	China	Pig	2016	98.57	OQ743896	/	/	/	/	/	/	/	HN03(I5)	MH021179.1	China	Pig	2018	98.41	OQ799750
CHN/LN/KJW1/2022	2022.1	Liaoning	TM-a-P60(G9)	MH697651.1	China	Pig	2018	97.25	OQ743852	GHA-14(P7)	MN102368.1	UK	Pig	2016	93.62	OQ799660	ZZ-12(I5)	KF303566.1	China	Pig	2013	97.49	OQ799754
CHN/LN/KJW4/2022	2022.1	Liaoning	TM-a-P60(G9)	MH697651.1	China	Pig	2018	97.45	OQ743853	GHA-14(P7)	MN102368.1	UK	Pig	2016	93.72	OQ799661	ZZ-12(I5)	KF303566.1	China	Pig	2013	97.40	OQ799755
CHN/SD/LB/2022	2022.1	Shandong	DZ-2(G5)	KT820775.1	China	Pig	2016	98.38	OQ743855	VNM-14175(P23)	KX363359.1	UK	Pig	2016	95.05	OQ799663	SCLS-3(I5)	MK597975.1	China	Pig	2018	96.82	OQ799757
CHN/SD/LC/2022	2022.1	Shandong	DZ-2(G5)	KT820775.1	China	Pig	2016	98.27	OQ743856	SWU-IC(P13)	MK410284.1	China	Pig	2018	97.30	OQ799664	HN03(I5)	MH021179.1	China	Pig	2018	97.74	OQ799758
CHN/SD/LC28/2022	2022.1	Shandong	SH0902(G1)	GU124595.1	China	Pig	2009	95.37	OQ743857	GHA-14(P7)	MN102368.1	UK	Pig	2016	93.89	OQ799665	/	/	/	/	/	/	/
CHN/GD/LG3/2022	2022.1	Guangdong	/	/	/	/	/	/	/	/	/	/	/	/	/	/	HN03(I5)	MH021179.1	China	Pig	2018	97.74	OQ799748
CHN/GD/LG5/2022	2022.1	Guangdong	/	/	/	/	/	/	/	/	/	/	/	/	/	/	ZZ-12(I5)	KF303566.1	China	Pig	2013	96.90	OQ799747
CHN/GX/MP4/2022	2022.1	Guangxi	LLP48(G9)	KJ126835.1	China	Pig	2018	97.36	OQ743858	HLJ/15(P23)	KU886316.1	China	Pig	2016	96.25	OQ799666	TA-3-1(I5)	KT820771.1	China	Pig	2016	96.65	OQ799759
CHN/GX/XS7/2022	2022.1	Guangxi	DZ-2(G5)	KT820775.1	China	Pig	2016	98.28	OQ743847	/	/	/	/	/	/	/	/	/	/	/	/	/	/

CHN/GD/XC2/2022	2022.1	Guangdong	/	/	/	/	/	/	/	/	/	/	/	/	/	/	HN03(I5)	MH021179.1	China	Pig	2018	97.57	OQ799752
CHN/GS/XC224/2022	2022.2	Ganus	CH-1(G1)	GU188284.1	China	Giant panda	2013	94.12	OQ743977	/	/	/	/	/	/	/	/	/	/	/	/	/	/
CHN/GS/XC5/2022	2022.2	Ganus	CH-1(G1)	GU188284.1	China	Giant panda	2013	94.22	OQ743980	/	/	/	/	/	/	/	/	/	/	/	/	/	/
CHN/GZ/KZM02/2022	2022.2	Guizhou	LL51695(G9)	KC242226.1	China	Human	2013	94.29	OQ743930	R1954(P6)	KF726067.1	China	Human	2013	96.61	OQ799674	R1954(I1)	KF726068.1	China	Human	2013	95.90	OQ799771
CHN/SD/LYXH/2022	2022.2	Shandong	SZ18(G4)	OM920725.1	China	Human	2018	96.83	OQ743863	R1954(P6)	KF726067.1	China	Human	2013	96.57	OQ799669	R946(I1)	KF726057.1	China	Human	2006	96.57	OQ799766
CHN/GX/DM4/2022	2022.2	Guangxi	HK69(G5)	JN699034.1	USA	Human	2013	93.27	OQ743867	SCYA-H2(P13)	MT198757.1	China	Pig	2019	96.78	OQ799675	VNM(I5)	LC095879.1	Japan	Human	2007	98.24	OQ799772
CHN/GZ/KZZD40/2022	2022.2	Guizhou	R1270(G4)	LC389890.1	SriLankan	Human	2009	96.53	OQ743928	/	/	/	/	/	/	/	VNM-NT0042(I1)	LC095890.1	Vietnam	Human	2018	96.15	OQ799779
CHN/HeN/LBZD02/2022	2022.2	Henan	Segment9(G9)	AY003871.1	China	Human	2004	94.70	OQ743864	HLJ/15(P23)	KU886316.1	China	Pig	2016	97.56	OQ799671	R479(I5)	DQ873675.1	China	Human	2007	96.15	OQ799768
CHN/GX/TL1/2022	2022.2	Guangxi	CU-B1738(G4)	KT007665.1	Thailand	Human	2013	96.32	OQ743860	GHA-14(P7)	MN102368.1	UK	Pig	2016	93.44	OQ799667	CMH-N016-10(I1)	MG781035.1	Thailand	Human	2010	96.73	OQ799762
CHN/GX/TL3/2022	2022.2	Guangxi	CU-B1738(G4)	KT007665.1	Thailand	Human	2013	96.84	OQ743859	GHA-14(P7)	MN102368.1	UK	Pig	2016	93.48	OQ799668	CMH-N016-10(I1)	MG781035.1	Thailand	Human	2010	96.73	OQ799763
CHN/GS/YM2/2022	2022.2	Gansu	243(G1)	MK050137.1	Iran	Human	2016	88.48	OQ743868	GHA-14(P7)	MN102368.1	UK	Pig	2016	94.01	OQ799677	RMC321(I5)	AF531913.1	India	Human	2004	95.48	OQ799774
CHN/HN/BP2/2022	2022.2	Hainan	LLP48(G9)	KJ126835.1	China	Pig	2018	97.07	OQ743861	/	/	/	/	/	/	/	R1954(I1)	KF726068.1	China	Human	2013	96.73	OQ799765
CHN/GZ/KZZD39/2022	2022.2	Guizhou	/	/	/	/	/	/	/	SCYA-H2(P13)	MT198757.1	China	Pig	2019	97.17	OQ799681	R479(I5)	DQ873675.1	China	Human	2007	95.81	OQ799778
CHN/SD/LYXL/2022	2022.2	Shandong	Arg720A(G12)	EU496257.1	Argentina	Human	2009	97.66	OQ743862	GHA-14(P7)	MN102368.1	UK	Pig	2016	93.26	OQ799670	WH-a(I5)	JN034041.1	China	Pig	2010	95.64	OQ799767
CHN/HeN/TY102/2022	2022.2	Henan	LLP48(G9)	KJ126835.1	China	Pig	2018	97.06	OQ743866	SCMY-A3(P23)	MK026438.1	China	Pig	2017	94.77	OQ799673	R479(I5)	DQ873675.1	China	Human	2007	96.23	OQ799770
CHN/HeN/TYZD02/2022	2022.2	Henan	TM-a-P60(G9)	MH697651.1	China	Pig	2018	97.15	OQ743865	SCMY-A3(P23)	MK026438.1	China	Pig	2017	94.77	OQ799672	R479(I5)	DQ873675.1	China	Human	2007	96.31	OQ799769
CHN/GX/TL4/2022	2022.2	Guangxi	/	/	/	/	/	/	/	/	/	/	/	/	/	/	R1954(I1)	KF726068.1	China	Human	2013	96.73	OQ799764
CHN/GX/WG2/2022	2022.2	Guangxi	LL51695(G9)	KC242226.1	China	Human	2013	94.01	OQ743931	/	/	/	/	/	/	/	/	/	/	/	/	/	/
CHN/GX/WG6/2022	2022.2	Guangxi	LL51695(G9)	KC242226.1	China	Human	2013	94.50	OQ743929	SCYB-C3(P23)	MT198752.1	China	Pig	2019	94.96	OQ799676	SWU-IC(I5)	MK410285.1	China	Pig	2018	98.91	OQ799773
CHN/GZ/BJ2/2022	2022.2	Guizhou	SWU-1C(G9)	MK410283.1	China	Pig	2018	95.52	OQ743870	SCYB-C3(P23)	MT198752.1	China	Pig	2019	97.69	OQ799678	SWU-IC(I5)	MK410285.1	China	Pig	2018	97.49	OQ799775
CHN/CY/GYSX/2022	2022.2	Sichuan	LLP48(G9)	KJ126835.1	China	Pig	2018	97.36	OQ743871	GHA-14(P7)	MN102368.1	UK	Pig	2016	94.59	OQ799679	ZZ-12(I5)	KF303566.1	China	Pig	2013	97.07	OQ799776

CHN/Shaanxi/HZ1/2022	2022.2	Shaanxi	/	/	/	/	/	/	/	/	/	/	/	/	/	/	HN03(I5)	MH021179.1	China	Pig	2018	97.65	OQ799760
CHN/Shaanxi/HZ2/2022	2022.2	Shaanxi	/	/	/	/	/	/	/	/	/	/	/	/	/	/	HN03(I5)	MH021179.1	China	Pig	2018	95.06	OQ799761
CHN/HuB/XZ3/2022	2022.2	Hubei	DZ-2(G5)	KT820775.1	China	Pig	2016	97.06	OQ743872	CMP178(P13)	DQ536362.1	Thailand	Pig	2008	96.64	OQ799680	HN03(I5)	MH021179.1	China	Pig	2018	98.24	OQ799777
CHN/Shaanxi/YJ/2022	2022.2	Shaanxi	HLJ-9(G9)	JX498949.1	China	Pig	2012	96.02	OQ743869	/	/	/	/	/	/	/	/	/	/	/	/	/	/
CHN/SD/LYXH2/2022	2022.3	Shandong	SZ18(G4)	OM920725.1	China	Human	2018	96.73	OQ743873	R1954(P6)	KF726067.1	China	Human	2013	96.71	OQ799682	R946(I1)	KF726057.1	China	Human	2006	96.73	OQ799780
CHN/CY/DY/2022	2022.3	Sichuan	HeN4(G4)	JX498957.1	China	Pig	2012	97.86	OQ743886	R1207(P6)	LC389888.1	Japan	Human	2018	97.15	OQ799691	R1954(I1)	KF726068.1	China	Human	2013	98.49	OQ799793
CHN/SD/LYXH3/2022	2022.3	Shandong	DZ-2(G5)	KT820775.1	China	Pig	2016	97.56	OQ743874	R1954(P6)	KF726067.1	China	Human	2013	96.53	OQ799683	R946(I1)	KF726057.1	China	Human	2006	96.65	OQ799781
CHN/GX/TL5/2022	2022.3	Guangxi	CMH-N016-10(G4)	MG781036.1	Thailand	Human	2010	96.57	OQ743880	GHA-14(P7)	MN102368.1	UK	Pig	2016	93.48	OQ799688	CMH-N016-10(I1)	MG781035.1	Thailand	Human	2010	96.90	OQ799788
CHN/GX/NN/2022	2022.3	Guangxi	Arg720A(G12)	EU496257.1	Argentina	Human	2009	97.66	OQ743881	/	/	/	/	/	/	/	/	/	/	/	/	/	/
CHN/GX/XS3/2022	2022.3	Guangxi	684VN(G9)	AB091778.1	Tokyo	Human	2002	95.24	OQ743895	/	/	/	/	/	/	/	SCLS-3(I5)	MK597975.1	China	Pig	2018	97.15	OQ799803
CHN/SD/CG/2022	2022.3	Shandong	TM-a-P60(G9)	MH697651.1	China	Pig	2018	97.04	OQ743875	/	/	/	/	/	/	/	JN-1(I5)	KT820768.1	China	Pig	2016	98.58	OQ799782
CHN/CY/SZ/2022	2022.3	Sichuan	HN03(G9)	KY649279.1	China	Pig	2015	97.66	OQ743883	CMP178(P13)	DQ536362.1	Thailand	Pig	2008	95.63	OQ799689	TA-4-1(I5)	KT820772.1	China	Pig	2016	96.48	OQ799790
CHN/CY/XP/2022	2022.3	Sichuan	HN03(G9)	KY649279.1	China	Pig	2015	97.45	OQ743882	/	/	/	/	/	/	/	TA-4-1(I5)	KT820772.1	China	Pig	2016	96.48	OQ799789
CHN/CY/JH/2022	2022.3	Sichuan	SCJY-11(G9)	MH910079.1	China	Pig	2017	98.06	OQ743889	SC11(P23)	MH624176.1	China	Pig	2017	97.15	OQ799693	VNM14150(I5)	KX363332.1	UK	Pig	2016	95.58	OQ799796
CHN/GS/LZ/2022	2022.3	Gansu	HN03(G9)	KY649279.1	China	Pig	2015	97.25	OQ743892	/	/	/	/	/	/	/	HN03(I5)	MH021179.1	China	Pig	2018	98.66	OQ799798
CHN/CY/LS/2022	2022.3	Sichuan	ESP(G9)	MZ643386.1	Spanish	Pig	2020	96.13	OQ743887	SWU-IC(P13)	MK410284.1	China	Pig	2018	97.00	OQ799692	HN03(I5)	MH021179.1	China	Pig	2018	98.41	OQ799794
CHN/SD/LCXH03/2022	2022.3	Shandong	SH0902(G1)	GU124595.1	China	Pig	2009	95.37	OQ743893	/	/	/	/	/	/	/	TA-3-1(I5)	KT820771.1	China	Pig	2016	97.32	OQ799799
CHN/GX/MP1/2022	2022.3	Guangxi	/	/	/	/	/	/	/	/	/	/	/	/	/	/	TA-3-1(I5)	KT820771.1	China	Pig	2016	96.82	OQ799785
CHN/GX/MP2/2022	2022.3	Guangxi	LLP48(G9)	KJ126835.1	China	Pig	2018	97.55	OQ743878	HLJ/15(P23)	KU886316.1	China	Pig	2016	96.26	OQ799686	TA-3-1(I5)	KT820771.1	China	Pig	2016	96.65	OQ799786
CHN/GX/MP3/2022	2022.3	Guangxi	LLP48(G9)	KJ126835.1	China	Pig	2018	97.36	OQ743876	HLJ/15(P23)	KU886316.1	China	Pig	2016	96.32	OQ799684	TA-3-1(I5)	KT820771.1	China	Pig	2016	96.65	OQ799783
CHN/GX/MP5/2022	2022.3	Guangxi	LLP48(G9)	KJ126835.1	China	Pig	2018	97.16	OQ743877	HLJ/15(P23)	KU886316.1	China	Pig	2016	96.20	OQ799685	TA-3-1(I5)	KT820771.1	China	Pig	2016	96.73	OQ799784
CHN/GX/MP6/2022	2022.3	Guangxi	SH0902(G1)	GU124595.1	China	Pig	2009	94.86	OQ743879	GHA-14(P7)	MN102368.1	UK	Pig	2016	93.70	OQ799687	SWU-IC(I5)	MK410285.1	China	Pig	2018	96.98	OQ799787
CHN/CY/QQ/2022	2022.3	Sichuan	SH0902(G1)	GU124595.1	China	Pig	2009	94.86	OQ743890	GHA-14(P7)	MN102368.1	UK	Pig	2016	94.23	OQ799694	TA-4-1(I5)	KT820772.1	China	Pig	2016	97.32	OQ799797
CHN/CY/SW/2022	2022.3	Sichuan	HN03(G9)	KY649279.1	China	Pig	2015	97.66	OQ743888	/	/	/	/	/	/	/	TA-4-1(I5)	KT820772.1	China	Pig	2016	96.57	OQ799795

CHN/LN/TW/2022	2022.3	Liaoning	TM-a-P60(G9)	MH697651.1	China	Pig	2018	97.15	OQ743891	/	/	/	/	/	/	/	/	/	/	/	/	/	/
CHN/GX/TL6/2022	2022.3	Guangxi	LLP48(G9)	KJ126835.1	China	Pig	2018	97.07	OQ743894	/	/	/	/	/	/	/	/	/	/	/	/	/	/
CHN/CY/TZ/2022	2022.3	Sichuan	SCQL-5-2(G3)	MG066590.1	China	Pig	2017	97.15	OQ743885	SCYA-H2(P13)	MT198757.1	China	Pig	2019	97.21	OQ799690	TA-4-1(I5)	KT820772.1	China	Pig	2016	96.23	OQ799792
CHN/CY/YX/2022	2022.3	Sichuan	HN03(G9)	KY649279.1	China	Pig	2015	97.45	OQ743884	/	/	/	/	/	/	/	HN03(I5)	MH021179.1	China	Pig	2018	97.82	OQ799791
CHN/HeN/LB2/2022	2022.4	Henan	SD-18(G9)	ON676185.1	China	Pig	2021	97.86	OQ743898	/	/	/	/	/	/	/	R479(I5)	DQ873675.1	China	Human	2007	96.57	OQ799800
CHN/HeN/TY204/2022	2022.4	Henan	/	/	/	/	/	/	/	/	/	/	/	/	/	/	R479(I5)	DQ873675.1	China	Human	2007	95.90	OQ799804
CHN/GS/XC6/2022	2022.4	Gansu	TM-a-P20(G9)	MH697629.1	China	Pig	2018	97.45	OQ743897	/	/	/	/	/	/	/	R479(I5)	DQ873675.1	China	Human	2007	96.40	OQ799801
CHN/ZJ/04/2022	2022.4	Zhejiang	/	/	/	/	/	/	/	Z84(P6)	MG570048.1	China	Pig	2007	95.87	OQ799695	R1207(I1)	LC389889.1	Japan	Human	2018	98.49	OQ799805
CHN/GZ/SB/2022	2022.4	Guizhou	/	/	/	/	/	/	/	/	/	/	/	/	/	/	SCLS-3(I5)	MK597975.1	China	Pig	2018	97.65	OQ799802
CHN/SD/CYFMD2/2022	2022.5	Shandong	CH-1(G1)	GU188284.1	China	Giant panda	2013	94.50	OQ743903	/	/	/	/	/	/	/	JN-1(I5)	KT820768.1	China	Pig	2016	98.49	OQ799814
CHN/HuB/Gl4/2022	2022.5	Hubei	Segment9(G9)	AY003871.1	China	Human	2004	94.76	OQ743914	HLJ/15(P23)	KU886316.1	China	Pig	2016	97.51	OQ799708	R479(I5)	DQ873675.1	China	Human	2007	96.30	OQ799825
CHN/HN/MY/2022	2022.5	Hainan	Segment9(G9)	AY003871.1	China	Human	2004	94.55	OQ743909	SWU-IC(P13)	MK410284.1	China	Pig	2018	96.88	OQ799700	VNM-30378(I5)	HG513050.1	Vietnam	Human	2014	97.08	OQ799810
CHN/CY/SX3/2022	2022.5	Sichuan	LL4260(G5)	EF159576.1	China	Human	2008	94.62	OQ743989	/	/	/	/	/	/	/	R1954(I1)	KF726068.1	China	Human	2013	98.27	OQ799879
CHN/CY/SXZD/2022	2022.5	Sichuan	/	/	/	/	/	/	/	R1207(P6)	LC389888.1	Japan	Human	2018	98.12	OQ799744	R1954(I1)	KF726068.1	China	Human	2013	98.27	OQ799878
CHN/HuB/Gl3/2022	2022.5	Hubei	SWU-1C(G9)	MK410283.1	China	Pig	2018	94.86	OQ743915	/	/	/	/	/	/	/	R479(I5)	DQ873675.1	China	Human	2007	96.47	OQ799824
CHN/CY/LH9/2022	2022.5	Sichuan	HLJxf(G9)	JX498950.1	China	Pig	2012	95.11	OQ743917	/	/	/	/	/	/	/	R479(I5)	DQ873675.1	China	Human	2007	96.19	OQ799827
CHN/Shaanxi/PC1/2022	2022.5	Shaanxi	LL51695(G9)	KC242226.1	China	Human	2013	94.70	OQ743901	NMTL(P23)	JF781161.1	China	Pig	2008	94.95	OQ799701	WF-1-1(I5)	KT820773.1	China	Pig	2016	96.31	OQ799811
CHN/Shaanxi/PC/2022	2022.5	Shaanxi	LL51695(G9)	KC242226.1	China	Human	2013	93.88	OQ743900	/	/	/	/	/	/	/	VNM14150(I5)	KX363332.1	UK	Pig	2016	94.82	OQ799808
CHN/GZ/DY/2022	2022.5	Guizhou	VP7(G26)	AB605258.1	Japan	Pig	2011	95.82	OQ743911	TWN-106(P3)	OL956956.1	China	Pig	2017	92.39	OQ799705	VNM14150(I5)	KX363332.1	UK	Pig	2016	95.73	OQ799821
CHN/GZ/KZMZD05/2022	2022.5	Guizhou	DZ-2(G5)	KT820775.1	China	Pig	2016	97.96	OQ743926	SCYA-H2(P13)	MT198757.1	China	Pig	2019	96.66	OQ799697	TA-3-1(I5)	KT820771.1	China	Pig	2016	96.49	OQ799807
CHN/GZ/KZM2/2022	2022.5	Guizhou	LLP48(G9)	KJ126835.1	China	Pig	2018	95.66	OQ743927	SCYA-H2(P13)	MT198757.1	China	Pig	2019	95.59	OQ799696	TA-3-1(I5)	KT820771.1	China	Pig	2016	96.49	OQ799806
CHN/CY/LH5/2022	2022.5	Sichuan	HLJxf(G9)	JX498950.1	China	Pig	2012	95.18	OQ743910	HLJ/15(P23)	KU886316.1	China	Pig	2016	97.01	OQ799698	JN-1(I5)	KT820768.1	China	Pig	2016	98.19	OQ799820
CHN/Shaanxi/PC4/2022	2022.5	Shaanxi	VP7(G5)	DQ515961.1	Thailand	Pig	2018	92.15	OQ743902	NMTL(P23)	JF781161.1	China	Pig	2008	94.91	OQ799702	TA-4-1(I5)	KT820772.1	China	Pig	2016	97.49	OQ799813

CHN/HeN/TQ/2022	2022.5	Henan	HN03(G9)	KY649279.1	China	Pig	2015	98.17	OQ743918	/	/	/	/	/	/	/	/	/	/	/	/	/	/
CHN/HeN/TYZD05/2022	2022.5	Henan	LLP48(G9)	KJ126835.1	China	Pig	2018	97.17	OQ743899	/	/	/	/	/	/	/	TA-3-1(I5)	KT820771.1	China	Pig	2016	96.99	OQ799819
CHN/Shanxi/XT1/2022	2022.5	Shanxi	LNCY(G3)	MF462326.1	China	Pig	2016	92.66	OQ743907	SCYA-H2(P13)	MT198757.1	China	Pig	2019	96.82	OQ799704	VNM14150(I5)	KX363332.1	UK	Pig	2016	95.40	OQ799818
CHN/Shanxi/XT118/2022	2022.5	Shanxi	NM-9(G9)	JX498952.1	China	Pig	2012	96.72	OQ743912	VP4(P23)	KC113250.1	China	Pig	2013	94.84	OQ799706	VNM14150(I5)	KX363332.1	UK	Pig	2016	95.48	OQ799822
CHN/Shanxi/XT3/2022	2022.5	Shanxi	/	/	/	/	/	/	/	SCYA-H2(P13)	MT198757.1	China	Pig	2019	96.79	OQ799699	VNM14150(I5)	KX363332.1	UK	Pig	2016	95.56	OQ799835
CHN/Shanxi/XT5/2022	2022.5	Shanxi	NM-9(G9)	JX498952.1	China	Pig	2012	96.74	OQ743906	/	/	/	/	/	/	/	VNM14150(I5)	KX363332.1	UK	Pig	2016	95.31	OQ799817
CHN/Shanxi/XT6/2022	2022.5	Shaanxi	LNCY(G3)	MF462326.1	China	Pig	2016	93.08	OQ743905	/	/	/	/	/	/	/	JN-1(I5)	KT820768.1	China	Pig	2016	98.16	OQ799816
CHN/Shanxi/XT7/2022	2022.5	Shanxi	LNCY(G3)	MF462326.1	China	Pig	2016	92.86	OQ743904	SCYA-H2(P13)	MT198757.1	China	Pig	2019	96.82	OQ799703	VNM14150(I5)	KX363332.1	UK	Pig	2016	95.64	OQ799815
CHN/Shanxi/XT8/2022	2022.5	Shanxi	/	/	/	/	/	/	/	/	/	/	/	/	/	/	JN-1-1(I5)	KT820767.1	China	Pig	2016	95.64	OQ799809
CHN/JS/XZ6/2022	2022.5	Jiangsu	HLJsh-1(G9)	JX498948.1	China	Pig	2012	96.43	OQ743916	/	/	/	/	/	/	/	HN03(I5)	MH021179.1	China	Pig	2018	97.77	OQ799826
CHN/CY/LH8/2022	2022.6	Sichuan	Segment9(G9)	AY003871.1	China	Human	2004	94.29	OQ743920	/	/	/	/	/	/	/	JN-1(I5)	KT820768.1	China	Pig	2016	98.51	OQ799829
CHN/Shanxi/XT818/2022	2022.6	Shanxi	CH-1(G1)	GU188284.1	China	Giant panda	2013	94.50	OQ743913	GHA-14(P7)	MN102368.1	UK	Pig	2016	93.91	OQ799707	VNM14150(I5)	KX363332.1	UK	Pig	2016	95.48	OQ799823
CHN/HuN/CT4/2022	2022.6	Hunan	VNM(G3)	KY021150.1	Vietnam	Pig	2017	96.70	OQ743919	MOZ(P13)	MT784813.1	Mozambique	Pig	2021	91.40	OQ799709	WF-1-1(I5)	KT820773.1	China	Pig	2016	96.93	OQ799828
CHN/ZJ/CH1/2022	2022.6	Zhejiang	GXqz-2(G9)	JX498942.1	China	Pig	2012	96.92	OQ743921	GHA-14(P7)	MN102368.1	UK	Pig	2016	93.21	OQ799711	TA-3-1(I5)	KT820771.1	China	Pig	2016	96.68	OQ799831
CHN/ZJ/CH2/2022	2022.6	Zhejiang	GXqz-2(G9)	JX498942.1	China	Pig	2012	97.06	OQ743925	GHA-14(P7)	MN102368.1	UK	Pig	2016	93.28	OQ799713	TA-3-1(I5)	KT820771.1	China	Pig	2016	96.90	OQ799830
CHN/ZJ/CH3/2022	2022.6	Zhejiang	GXqz-2(G9)	JX498942.1	China	Pig	2012	97.26	OQ743922	GHA-14(P7)	MN102368.1	UK	Pig	2016	93.38	OQ799712	TA-3-1(I5)	KT820771.1	China	Pig	2016	96.90	OQ799833
CHN/JS/JR/2022	2022.6	Jiangsu	VP7(G26)	AB605258.1	Japan	pig	2011	96.53	OQ743987	/	/	/	/	/	/	/	/	/	/	/	/	/	/
CHN/LN/KJW2/2022	2022.6	Liaoning	DZ-2(G5)	KT820775.1	China	Pig	2016	97.86	OQ743983	SCYA-H2(P13)	MT198757.1	China	Pig	2019	96.80	OQ799741	TA-3-1(I5)	KT820771.1	China	Pig	2016	97.09	OQ799875
CHN/LN/KJW2/96/2022	2022.6	Liaoning	HLJxf(G9)	JX498950.1	China	Pig	2012	94.22	OQ743984	/	/	/	/	/	/	/	/	/	/	/	/	/	/
CHN/LN/KJW411/2022	2022.6	Liaoning	DZ-2(G5)	KT820775.1	China	Pig	2016	97.97	OQ743985	SCYA-H2(P13)	MT198757.1	China	Pig	2019	96.81	OQ799742	TA-3-1(I5)	KT820771.1	China	Pig	2016	97.01	OQ799876
CHN/HeN/LB106/2022	2022.6	Henan	NM-9(G9)	JX498952.1	China	Pig	2012	94.49	OQ743924	HLJ/15(P23)	KU886316.1	China	Pig	2016	97.40	OQ799710	HN03(I5)	MH021179.1	China	Pig	2018	98.24	OQ799834
CHN/HeN/LB206/2022	2022.6	Henan	NM-9(G9)	JX498952.1	China	Pig	2012	94.57	OQ743923	/	/	/	/	/	/	/	HN03(I5)	MH021179.1	China	Pig	2018	98.39	OQ799832
CHN/GS/XC1/2022	2022.6	Gansu	TM-a-P20(G9)	MH697629.1	China	Pig	2018	97.25	OQ743962	/	/	/	/	/	/	/	/	/	/	/	/	/	/
CHN/GS/XC2/2022	2022.6	Gansu	TM-a-P20(G9)	MH697629.1	China	Pig	2018	97.15	OQ743975	/	/	/	/	/	/	/	/	/	/	/	/	/	/

CHN/GS/XC4/2022	2022.6	Gansu	TM-a-P20(G9)	MH697629.1	China	Pig	2018	97.55	OQ743979	/	/	/	/	/	/	/	/	/	/	/	/	/	/
CHN/ZJ/XH2/2022	2022.6	Zhejiang	LLP48(G9)	KJ126835.1	China	Pig	2018	95.54	OQ743986	/	/	/	/	/	/	/	/	/	/	/	/	/	/
CHN/ZJ/XH3/2022	2022.6	Zhejiang	LLP48(G9)	KJ126835.1	China	Pig	2018	95.33	OQ743982	SCYA-C7(P13)	MT198756.1	China	pig	2019	93.97	OQ799740	/	/	/	/	/	/	/
CHN/JS/XZ/2022	2022.6	Jiangsu	HLJsh-1(G9)	JX498948.1	China	Pig	2012	96.24	OQ743940	CMP178(P13)	DQ536362.1	Thailand	Pig	2008	95.97	OQ799717	HN03(I5)	MH021179.1	China	Pig	2018	97.64	OQ799842
CHN/ZJ/ZJ1/2022	2022.6	Zhejiang	LLP48(G9)	KJ126835.1	China	Pig	2018	95.33	OQ743947	SCYA-C7(P13)	MT198756.1	China	pig	2019	94.88	OQ799721	/	/	/	/	/	/	/
CHN/ZJ/ZYF6/2022	2022.6	Zhejiang	34461-4(G2)	AY766085.1	Italy	pig	2005	87.74	OQ743958	/	/	/	/	/	/	/	/	/	/	/	/	/	/
CHN/GD/DD1/2022	2022.7	Guangdong	DZ-2(G5)	KT820775.1	China	Pig	2016	97.16	OQ743950	FX17(P13)	OM362094.1	China	pig	2022	97.79	OQ799722	HN03(I5)	MH021179.1	China	Pig	2018	98.35	OQ799850
CHN/HN/FH2/2022	2022.7	Hainan	LLP48(G9)	KJ126835.1	China	Pig	2018	97.46	OQ743932	/	/	/	/	/	/	/	TA-3-1(I5)	KT820771.1	China	Pig	2016	96.86	OQ799836
CHN/HN/FH3/2022	2022.7	Hainan	LLP48(G9)	KJ126835.1	China	Pig	2018	97.46	OQ743935	/	/	/	/	/	/	/	/	/	/	/	/	/	/
CHN/SD/LP1/2022	2022.7	Shandong	LLP48(G9)	KJ126835.1	China	Pig	2018	97.57	OQ743933	/	/	/	/	/	/	/	TA-3-1(I5)	KT820771.1	China	Pig	2016	96.86	OQ799837
CHN/SD/LP3/2022	2022.7	Shandong	LLP48(G9)	KJ126835.1	China	Pig	2018	97.26	OQ743936	FX17(P13)	OM362094.1	China	pig	2022	97.71	OQ799714	TA-3-1(I5)	KT820771.1	China	Pig	2016	96.70	OQ799838
CHN/GD/LG2/2022	2022.7	Guangdong	VP7(G26)	AB605258.1	Japan	pig	2011	94.29	OQ743954	/	/	/	/	/	/	/	TA-3-1(I5)	KT820771.1	China	Pig	2016	96.78	OQ799852
CHN/LN/TT1/2022	2022.7	Liaoning	HLJxf(G9)	JX498950.1	China	Pig	2012	95.03	OQ743948	/	/	/	/	/	/	/	WF-1-1(I5)	KT820773.1	China	Pig	2016	96.47	OQ799849
CHN/LN/TT3/2022	2022.7	Liaoning	DZ-2(G5)	KT820775.1	China	Pig	2016	98.28	OQ743945	HLJ/15(P23)	KU886316.1	China	Pig	2016	94.31	OQ799719	WF-1-1(I5)	KT820773.1	China	Pig	2016	96.55	OQ799847
CHN/LN/TT7/2022	2022.7	Liaoning	HLJxf(G9)	JX498950.1	China	Pig	2012	95.23	OQ743966	/	/	/	/	/	/	/	JN-1(I5)	KT820768.1	China	Pig	2016	98.42	OQ799863
CHN/LN/TTZD/2022	2022.7	Liaoning	DZ-2(G5)	KT820775.1	China	Pig	2016	98.27	OQ743981	HLJ/15(P23)	KU886316.1	China	Pig	2016	94.14	OQ799739	JN-1(I5)	KT820768.1	China	Pig	2016	97.16	OQ799874
CHN/SD/CL3/2022	2022.8	Shandong	LLP48(G9)	KJ126835.1	China	Pig	2018	97.57	OQ743934	/	/	/	/	/	/	/	/	/	/	/	/	/	/
CHN/GZ/GX3/2022	2022.8	Guizhou	DZ-2(G5)	KT820775.1	China	Pig	2016	98.17	OQ743965	/	/	/	/	/	/	/	VP6(I5)	KC113249.1	China	pig	2013	96.64	OQ799862
CHN/TJ/HX5/2022	2022.8	Tianjin	VP7(G26)	AB605258.1	Japan	pig	2011	96.33	OQ743972	SCYA-H2(P13)	MT198757.1	China	Pig	2019	97.21	OQ799735	HBTS96(I5)	OM735819.1	China	pig	2021	98.49	OQ799869
CHN/HeN/LB1/2022	2022.8	Henan	VP7(G5)	DQ515961.1	Thailand	Pig	2018	92.20	OQ743974	CMP178(P13)	DQ536362.1	Thailand	Pig	2008	96.24	OQ799736	HN03(I5)	MH021179.1	China	Pig	2018	98.44	OQ799871
CHN/FJ/LH/2022	2022.8	Fujian	DZ-2(G5)	KT820775.1	China	Pig	2016	97.87	OQ743961	/	/	/	/	/	/	/	TA-3-1(I5)	KT820771.1	China	Pig	2016	97.09	OQ799858
CHN/HeN/TQ2/2022	2022.8	Henan	SD-1-2021(G9)	ON676185.1	China	pig	2021	97.55	OQ743943	/	/	/	/	/	/	/	JN-1(I5)	KT820768.1	China	Pig	2016	98.58	OQ799845
CHN/SD/LC2/2022	2022.9	Shandong	SCCD-A(G9)	MH910068.1	China	Dog	2017	97.04	OQ743951	/	/	/	/	/	/	/	/	/	/	/	/	/	/
CHN/SD/LCYGZD/2022	2022.9	Shandong	SCCD-A(G9)	MH910068.1	China	Dog	2017	96.94	OQ743953	FX17(P13)	OM362094.1	China	pig	2022	97.79	OQ799723	HN03(I5)	MH021179.1	China	Pig	2018	97.64	OQ799851

CHN/ZJ/JH/2022	2022.9	Zhejiang	CH-1(G1)	GU188284.1	China	Giant panda	2013	94.39	OQ743908	/	/	/	/	/	/	/	TA-4-1(I5)	KT820772.1	China	Pig	2016	96.49	OQ799812
CHN/ZJ/ZJ2/2022	2022.9	Zhejiang	CH-1(G1)	GU188284.1	China	Giant panda	2013	94.12	OQ743949	/	/	/	/	/	/	/	/	/	/	/	/	/	/
CHN/GS/ZC2/2022	2022.9	Gansu	CH-1(G1)	GU188284.1	China	Giant panda	2013	94.31	OQ743988	GHA-14(P7)	MN102368.1	UK	Pig	2016	93.84	OQ799743	HN03(I5)	MH021179.1	China	Pig	2018	98.35	OQ799877
CHN/GS/ZC3/2022	2022.9	Gansu	CH-1(G1)	GU188284.1	China	Giant panda	2013	94.32	OQ743957	/	/	/	/	/	/	/	/	/	/	/	/	/	/
CHN/SD/DE/2022	2022.9	Shandong	LLP48(G9)	KJ126835.1	China	Pig	2018	97.36	OQ743941	/	/	/	/	/	/	/	HN03(I5)	MH021179.1	China	Pig	2018	98.19	OQ799843
CHN/LN/GJ3/2022	2022.9	Liaoning	VNM14250(G4)	KX363437.1	UK	pig	2016	96.14	OQ743968	GUB88(P6)	AB573872.1	Japan	Pig	2013	95.24	OQ799732	WF-1-1(I5)	KT820773.1	China	Pig	2016	97.49	OQ799865
CHN/LN/GJZD/2022	2022.9	Liaoning	DZ-2(G5)	KT820775.1	China	Pig	2016	98.88	OQ743970	VNM14150(P13)	KX363348.1	Britain	pig	2016	96.86	OQ799733	WF-1-1(I5)	KT820773.1	China	Pig	2016	97.49	OQ799867
CHN/SD/GT1/2022	2022.9	Shandong	VP7(G5)	DQ515961.1	Thailand	Pig	2018	92.50	OQ743963	SCLS18(P13)	MT198749.1	China	pig	2019	94.72	OQ799728	HeNNY-01(I5)	MW575221.1	China	pig	2022	97.41	OQ799859
CHN/SD/GT3/2022	2022.9	Shandong	VP7(G5)	DQ515961.1	Thailand	Pig	2018	92.40	OQ743964	SCLS18(P13)	MT198749.1	China	pig	2019	94.59	OQ799730	HN03(I5)	MH021179.1	China	Pig	2018	98.19	OQ799861
CHN/JX/JS1/2022	2022.9	Jiangxi	VP7(G26)	AB605258.1	Japan	pig	2011	94.60	OQ743991	LNCY(P13)	MF462324.1	China	pig	2016	97.79	OQ799727	TA-3-1(I5)	KT820771.1	China	Pig	2016	96.54	OQ799857
CHN/JX/JS2/2022	2022.9	Jiangxi	/	/	/	/	/	/	/	MOZ(P13)	MT784812.1	Mozambique	Pig	2021	91.82	OQ799729	HN03(I5)	MH021179.1	China	Pig	2018	97.41	OQ799860
CHN/CY/SX3/P6/2022	2022.9	Sichuan	HLJhg7(G11)	JX498964.1	China	Pig	2012	95.33	OQ743990	SCYA-H2(P13)	MT198757.1	China	Pig	2019	97.21	OQ799745	/	/	/	/	/	/	/
CHN/SD/LYTGZ3/2022	2022.9	Shandong	LY-2(G9)	KT820778.1	China	pig	2015	96.35	OQ743955	/	/	/	/	/	/	/	HN03(I5)	MH021179.1	China	Pig	2018	97.56	OQ799854
CHN/NM/TL12/2022	2022.9	Inner Mongolia	TWN103(G9)	KU739974.1	Taiwan-China	pig	2017	93.42	OQ743978	/	/	/	/	/	/	/	TA-3-1(I5)	KT820771.1	China	Pig	2016	97.33	OQ799873
CHN/LN/YTW1/2022	2022.9	Liaoning	SD-1-2021(G9)	ON676185.1	China	pig	2021	97.35	OQ743944	/	/	/	/	/	/	/	JN-1(I5)	KT820768.1	China	Pig	2016	98.51	OQ799846
CHN/LN/YTW2/2022	2022.9	Liaoning	TM-a-P60(G9)	MH697651.1	China	Pig	2018	97.55	OQ743946	HLJ/15(P23)	KU886316.1	China	Pig	2016	94.61	OQ799720	JN-1(I5)	KT820768.1	China	Pig	2016	98.58	OQ799848
CHN/SD/DJY6/2022	2022.1	Shandong	HuBEL2009(G9)	JQ993318.1	Belgium	Human	2012	94.62	OQ743967	MOZ(P13)	MT784812.1	Mozambique	Pig	2021	91.83	OQ799731	DZ-1(I5)	KY820766.1	China	pig	2016	95.68	OQ799864
CHN/HuB/GL2/2022	2022.1	Hubei	/	/	/	/	/	/	/	NMTL(P23)	JF781161.1	China	Pig	2008	97.44	OQ799718	R479(I5)	DQ873675.1	China	Human	2007	96.46	OQ799844
CHN/GD/WY1/2022	2022.1	Guangdong	D152(G9)	KU740013.1	Taiwan-China	Human	2015	93.94	OQ743956	CMP178(P13)	DQ536362.1	Thailand	Pig	2008	96.37	OQ799725	HN03(I5)	MH021179.1	China	Pig	2018	97.72	OQ799855
CHN/SD/CY3/2022	2022.1	Shandong	VP7(G26)	AB605258.1	Japan	pig	2011	94.80	OQ743960	/	/	/	/	/	/	/	JN-1(I5)	KT820768.1	China	Pig	2016	98.19	OQ799856

CHN/SD/NX2/2022	2022.1	Shandong	TM-a-P60(G9)	MH697651.1	China	Pig	2018	96.94	OQ743969	/	/	/	/	/	/	/	JN-1(I5)	KT820768.1	China	Pig	2016	98.66	OQ799866
CHN/SD/NX5/2022	2022.1	Shandong	TM-a-P60(G9)	MH697651.1	China	Pig	2018	96.94	OQ743971	CN127(P7)	ON989016.1	China	pig	2021	97.36	OQ799734	JN-1(I5)	KT820768.1	China	Pig	2016	98.66	OQ799868
CHN/SD/NX6/2022	2022.1	Shandong	TM-a-P60(G9)	MH697651.1	China	Pig	2018	96.94	OQ743973	CN127(P7)	ON989016.1	China	pig	2021	97.30	OQ799737	JN-1(I5)	KT820768.1	China	Pig	2016	98.66	OQ799870
CHN/SD/NX9/2022	2022.1	Shandong	VP7(G26)	AB605258.1	Japan	pig	2011	96.33	OQ743976	FX17(P13)	OM362094.1	China	pig	2022	97.86	OQ799738	HBTS96(I5)	OM735819.1	China	pig	2021	98.24	OQ799872
CHN/SD/TW/2022	2022.1	Shandong	VP7(G26)	AB605258.1	Japan	pig	2011	96.21	OQ743938	/	/	/	/	/	/	/	/	/	/	/	/	/	/
CHN/GZ/BJ1/2022	2022.1	Guizhou	LY-2(G9)	KT820778.1	China	pig	2015	95.63	OQ743937	MOZ(P13)	MT784812.1	Mozambique	Pig	2021	95.92	OQ799715	HeNNY-01(I5)	MW575221.1	China	pig	2022	97.17	OQ799839
CHN/TJ/HX6/2022	2022.1	Tianjin	VP7(G26)	AB605258.1	Japan	pig	2011	96.13	OQ743959	SCYA-H2(P13)	MT198757.1	China	Pig	2019	97.07	OQ799726	/	/	/	/	/	/	/
CHN/TJ/NHS6/2022	2022.1	Tianjin	VP7(G26)	AB605258.1	Japan	pig	2011	96.23	OQ743952	SCYA-H2(P13)	MT198757.1	China	Pig	2019	97.10	OQ799724	VNM14150(I5)	KX363332.1	UK	Pig	2016	96.62	OQ799853
CHN/SD/SX/2022	2022.1	Shandong	TM-a-P20(G9)	MH697629.1	China	Pig	2018	97.25	OQ743942	/	/	/	/	/	/	/	HBTS96(I5)	OM735819.1	China	pig	2021	98.24	OQ799840
CHN/CY/XP27/2022	2022.1	Sichuan	SCJY-11(G9)	MH910079.1	China	Pig	2017	98.67	OQ743939	NMTL(P23)	JF781161.1	China	Pig	2008	98.59	OQ799716	SC11(I5)	MH624177.1	China	pig	2017	97.57	OQ799841
