# Supplementary material for: Recent Molecular Characterization of Porcine Rotaviruses Detected in China and Their Phylogenetic Relationships with Human Rotaviruses
Source: Viruses. 2024 Mar 14;16(3):453. doi: 10.3390/v16030453 (PMC10975774; doi:10.3390/v16030453)
Supplement: Supplementary file 1 [file viruses-16-00453-s001.zip › Supplementary Materials Table S3.pdf]

Table S3. Whole genomic analyses of CHN/SD/LYXH2/2022/G4P6I1 strain detected in diarrheal piglet

Gene of LYXH2	Accession NO. of LYXH2	Closely related strain	Nucleotide homology (%)	Amino acid homology (%)	Genotype	Accession NO. of reference sequence
VP1	OQ799880	RVA/S79	95.71	96.9	R1	OM201230.1
VP2	OQ799881	RVA/Human/LL3354	95.51	87.8	C1	KC139782.1
VP3	OQ799882	RVA/pig-tc/SCMY/2017/G9P[23]	97.49	93.1	M1	MK026437.1
VP4	OQ799682	RVA/Human-wt/R1954/2013/G4P[6]	96.67	94.5	P6	KF726067.1
VP6	OQ799780	RVA/Human-wt/R946/2006/G3P[6]	96.73	100	I1	KF726057.1
VP7	OQ743873	RVA/Human-wt/SZ18-2049/2018/G4P[6]	96.73	95	G4	OM920725.1
NSP1	OQ799883	RVA/pig-wt/SCLSHL/2017/G9P[23]	95.82	83.9	A8	MH137274.1
NSP2	OQ799884	RVA/Human/LL3354	95.86	93.5	N1	KC139786.1
NSP3	OQ799885	RVA/pig-wt/MRC3878/2008/G5P[X]	97.03	90.6	T1	KP753059.1
NSP4	OQ799886	RVA/pig-wt/CN127/2021/G12P[7]	98.07	97.3	E1	ON989011.1
NSP5	OQ799887	RVA/Human/LL3354	98.49	99.3	H1	KC139789.1
