# Supplementary material for: Recent Molecular Characterization of Porcine Rotaviruses Detected in China and Their Phylogenetic Relationships with Human Rotaviruses
Source: Viruses. 2024 Mar 14;16(3):453. doi: 10.3390/v16030453 (PMC10975774; doi:10.3390/v16030453)
Supplement: Supplementary file 1 [file viruses-16-00453-s001.zip › Supplementary Materials Table S1.pdf]

Table S1 Primers used for PoRVA detection and genotyping in this study

Primer	Sequence	Size (bp)
qNSP3-F	5’-ACCATCTACACATGACCCTCTATGAG-3’	
qNSP3-R	5’-ACATAACGCCCCTATAGCCATTTAG-3’	83
qNSP3-P	5’-FAM-ACAATAGTTAAAAGCTAACACTG-BHQ1-3’	
VP7-F	5’-GGCTTTAAAAGAGAGAATTTCC-3’	1000
VP7-R	5’-GGTCACATCATACAATTCTAA-3’	
VP6-F	5’-GGCTTTWAAACGAAGTCTTC-3’	1356
VP6-R	5’-GGTCACATCCTCTCACTA-3’	
VP4-F	5’-GGCTATAAAATGGCTTCGCTCA-3’	2362
VP4-R	5’-TRCTTAYARTCTACATTGCA-3’	
VP1-F	5’-GGCTATTAAAGCTGTACAATGG-3’	3302
VP1-R	5’-GGTCACATCTAAGCRCTC-3’	
VP2-F	5’-GGCTATTAAAGGYTCAATGG-3’	2717
VP2-R	5’-GGTCATATCTCCACAGTGG-3’	
VP3-F	5’-ATGAAAGTATTAGCTTTAAG-3’	2525
VP3-R	5’-GTGTGTTAAGTTTTTAGCTCAC-3’	
NSP1-F	5’-ATGAAAAGTCTTGTRGAAGCC-3’	1500
NSP1-R	5’-CCTAGGCGCTACTCTAGTGC-3’	
NSP2-F	5’-GAGCCTTGCGGTGTAGCCATG-3’	992
NSP2-R	5’-GGTCACATAAGCGCTTTCT-3’	
NSP3-F	5’-GATGGAGTCTACTCAGCAGATGG-3’	933
NSP3-R	5’-CTATTGTGCTCATAGAGGGTC-3’	
NSP4-F	5’-AGTTCTGTTCCGAGAGAGCG-3’	723
NSP4-R	5’-TTCCTTCCATTAACGTCCAAC-3’	
NSP5-F	5’-GGCTTTTAAAGCGCTACAGTG-3’	609
NSP5-R	5’-ATCTTCGATCAATTGCATTGC-3’	

Note for degenerate bases: W=A or T; R=A or G; Y=C or T.
